# Reliability and validity of the Patient Experiences Questionnaire for Interdisciplinary Treatment for Substance Dependence – Continuous Electronic Measurement (PEQ-ITSD – CEM)

**DOI:** 10.1186/s12913-023-10506-7

**Published:** 2024-01-04

**Authors:** Hilde Hestad Iversen, Mona Haugum, Lina Harvold Ellingsen-Dalskau, Oyvind Bjertnaes

**Affiliations:** Norwegian Institute of Public Health, PO Box 222 Skoyen, Oslo, 0213 Norway

**Keywords:** Questionnaire, Patient experiences, Interdisciplinary treatment substance dependence, Substance use disorders, Validation, Psychometrics, Reliability, Validity

## Abstract

**Background:**

Inpatient experiences with interdisciplinary treatment for substance dependence and mental health care are measured using continuous electronic measurements in Norway. Major changes in data collection from cross-sectional surveys to continuous measurements necessitated the revalidation of the instrument. The main purpose of the present study was to determine the psychometric properties of the Patient Experiences Questionnaire for Interdisciplinary Treatment for Substance Dependence – Continuous Electronic Measurement (PEQ-ITSD – CEM). We also aimed to develop a short version of this tool, since completing the original version can be burdensome for some patients.

**Methods:**

The study included adult inpatients (aged ≥ 16 years) who received substance-dependence treatment at 102 different sections in Norway during 2020–2022 (*n* = 2,850). Factor structure and item performance were assessed. A short version was developed based on the psychometric testing results that included item response theory analysis.

**Results:**

The PEQ-ITSD – CEM comprised three empirically based scales with good internal consistency, reliability and validity, which covers treatment and personnel (14 items), milieu (6 items) and outcome (5 items). The results supported a seven-item short version, with three items selected for the treatment and personnel scale, two items for the milieu scale and two items for the outcome scale.

**Conclusions:**

The PEQ-ITSD – CEM can be recommended for future assessments of patient experiences with interdisciplinary treatment for substance dependence in Norway and in other countries with similar healthcare systems. This short-form version can be applied when respondent burden is a crucial issue.

**Supplementary Information:**

The online version contains supplementary material available at 10.1186/s12913-023-10506-7.

## Background

Patient experiences have become a key indicator of quality of healthcare and patient centeredness [1, 2], and are associated with treatment adherence, clinical outcomes and patient safety [2, 3]. Questionnaires are often used to obtain insight into patient experiences because of their capability to elicit responses from a large number of patients in a standardized manner [1]. Instrument choice should be determined by considering different utility aspects such as cost, acceptability by the users and educational impact [4]. When selecting an instrument, it is also crucial to thoroughly assess whether the instrument aligns with the specific objectives and content areas it is intended to measure, and if it provides meaningful and actionable results. Questionnaires that capture different aspects are essential to evaluate patient experiences with care, and should be developed with extensive involvement of users applying techniques such as interviews, focus groups and usability tests, and must be useful in guiding the quality improvement of clinical practice [5].

Care quality for patients with mental disorders has not improved to the same degree as for those with physical conditions, and the development and application of mental-health-care quality measures have lagged behind other areas of medicine [6]. The persistent gap in the quality of mental health care is partly due to the lack of systematic methods for measuring quality, and there are calls to include patient experiences in the applied quality measures, particularly their views about system structures, the care they have received and self-reported outcomes [6–8]. Substance use disorders is an umbrella term for the use of, and dependency on, drugs and alcohol, and are prevalent mental health issues in affluent countries. These disorders frequently coexist with other mental and physical ailments, present treatment challenges, and exert considerable social and economic strain on both close communities and society overall [9, 10]. The use of alcohol and illicit drugs is one of the most important risk factors for death in those younger than 70 years in Norway [9]. However, there is inadequate empirical evidence on the impact of interventions to treat substance use disorders, and a recent review highlighted the lack of examinations of patient experiences in relation to outcomes in these populations and the need for accessible and psychometrically validated patient-experience measures [11]. The review found an association between the patient-centred approach and outcomes, but its conclusions were impaired by the underrepresentation of patient-reported experience measures (PREMs). In contrast to satisfaction measures, PREMs focus on factual questions about whether certain processes and events occurred and are therefore more valuable in identifying problems with specific processes that impact the quality of care delivery [11, 12]. However, satisfaction is an important dimension of patient experience and is frequently assessed using specific items or even separate scales within a broader PREM instrument [2, 4].

The Norwegian Institute of Public Health (NIPH) is responsible for national patient-experience surveys in Norway. The purpose of the program is to systematically measure user experiences of health care as a basis for quality improvement, health care management, patient choice and public accountability. The standard multi-step process begins with identifying phenomena crucial to specific patient groups. Four aspects significantly shaped the experiences of patients undergoing substance dependence treatment. Patients stressed the importance of a welcoming and secure treatment setting. Respectful treatment and the feeling of being taken seriously by healthcare providers were highly valued, fostering trust and collaboration. Additionally, patients emphasize the importance of readily available staff and programs tailored to their individual needs, ensuring personalized treatment and support. Assistance in the context of preparations and planning for discharge and close follow-up post-discharge were seen as crucial for a successful transition and sustained recovery. These phenomena are then operationalized into multi-item scales, which are rigorously tested for reliability and validity using psychometric methods. The Patient Experiences Questionnaire for Interdisciplinary Treatment for Substance Dependence (PEQ-ITSD) was developed and tested using rigorous and comprehensive methods, and was applied in national cross-sectional surveys in 2013, 2014, 2015 and 2017.

The surveys were conducted on-site and included patients at different phases of their treatment. The PEQ-ITSD was specifically designed to assess patient experiences within inpatient or residential treatment settings for Substance Use Disorders (SUD), and not intended to cover the broader spectrum of SUD treatment modalities. Psychometric testing showed that the PEQ-ITSD had satisfactory internal consistency reliability and construct validity [13]. In 2019, the Ministry of Health and Care Services of Norway decided that from 2020 the experience surveys for inpatients who receive treatment for substance dependence and mental health care should be conducted using continuous electronic measurements. The surveys were still conducted on-site, but as close to the time of discharge as possible. The innovations aimed to improve the use of data from PREMs at the system level by integrating them into a multidimensional performance evaluation system, and at the individual ward level by supporting the adoption of patient-reported experiences in quality improvement work by health-care professionals. The previously validated measures of the PEQ-ITSD and the Psychiatric Inpatient Patient Experience Questionnaire – On Site were applied [13, 14].

Major changes in data collection from cross-sectional surveys to continuous measurements necessitated the revalidation of the two instruments. When the surveys were carried out using a cross-sectional design, patients were asked for their experiences in different treatment phases, to which they responded on a paper questionnaire. In the current measurements, patients are invited to reply using an electronic version of the questionnaire a few days before discharge.

A recent study determined the reliability and validity of the Psychiatric Inpatient Patient Experience Questionnaire – Continuous Electronic Measurement (PIPEQ – CEM) [15]. Employees at the psychiatric institutions that recruited patients to this national survey emphasized the need for a shorter questionnaire, and an objective of the psychometric testing was therefore to develop a short version of the PIPEQ – CEM for future application. The same concern was not raised by employees at residential treatment institutions for substance dependence. However, short versions have become increasingly more relevant since an increasing number of national surveys will be carried out as longitudinal studies in the future, and other instruments such as patient-reported outcomes and experiences with community services and collaboration across health-care levels need to be included. Short versions of the instruments are therefore relevant for the continuous electronic measurements and for various similar applications in Norway and other countries.

Most validation studies related to patient experiences of mental health care apply classical test theory (CTT) analysis [16]. The psychometric testing in the national survey program of Norway has until recently applied CTT. However, modern test theory provides richer and more accurate descriptions of performance at the item and scale levels and can increase the precision and standardization of measures while reducing the burden on the respondents [17]. Item response theory (IRT) has provided useful information that can be used to shorten instruments in recent studies [15, 18].

The aim of the present study was to determine the data quality, validity and internal consistency reliability of the Patient Experiences Questionnaire for Interdisciplinary Treatment for Substance Dependence – Continuous Electronic Measurement (PEQ-ITSD – CEM), which is currently used for large-scale measurements in Norway. The secondary objectives were to supplement classical psychometric methods using IRT to provide more information on item performance, and develop a short version of the instrument to reduce its burden on respondents.

## Methods

### Questionnaire development and content

The PEQ-ITSD – CEM, which is based on the PEQ-ITSD, is applied to perform continuous electronic measurements in Norway. The development and validation of the instrument followed the standard methodology to ensure sound psychometric properties, including a literature review, patient interviews, expert-group consultations and pilot testing [13]. The PEQ-ITSD – CEM was adapted to the current developments in data collection, in which patients are included close to the time of discharge. Small adjustments were made to the use of the “not applicable” response in some items (13, 31 and 35), since questions about preparation for the time after discharge were considered more relevant than in a cross-sectional setting. One question pertaining to whether the institution had provided satisfactory guidance and training on substance abuse issues was excluded, as the Norwegian Directorate of Health no longer requested information on this topic. A question regarding the patient's country of birth was added to the questionnaire. Additional file 1 presents the questionnaire.

The PEQ-ITSD – CEM consists of 58 closed-ended items, and two open-ended questions that ask the respondents to write more about their experiences at the institution, and about the help and care they had received from their municipality. The items on patient experiences are divided into different sections that address reception and waiting times (items 3, 4), therapists and staff (items 6–11), treatment (items 12–24), environment and activities (items 25–30), preparation for the time after discharge (items 31–34), other assessments (items 35–39, 41), previous admissions (item 43), previous help from the municipality (items 45–48) and individual plan for treatment/care (item 56). Six of these items are included at the request of the Norwegian Directorate of Health to monitor patient experiences in the context of treatment pathways for substance abuse (items 17, 18, 23, 24, 28 and 45). It should be noted that these six questions are not part of the core measurement instrument. Background questions include the drug/substance most frequently used prior to admission (item 1), length of stay (item 2), waiting time for admission (item 5), feeling pressured/forced to be admitted (item 40), previous admissions (items 42 and 44) and individual plan for treatment/care (item 55). The questionnaire also includes items on sociodemographic characteristics (items 49–54), and self-perceived mental and physical health (items 57 and 58).

Most of the items on experiences have a five-point response format, including the following response options; 1 = “not at all”, 2 = “to a small extent”, 3 = “to some extent”, 4 = “to a large extent”, and 5 = “to a very large extent”, with an additional response option of “not applicable”. The current response scale is consistently applied in surveys conducted by the NIPH, which makes it possible to compare responses over time and between different health-care user groups [13, 14, 19].

### Data collection

The survey was commissioned by the Ministry of Health and Care Services of Norway and was mandatory for all relevant institutions. Four regional health authorities are responsible for the specialist treatment of drug and alcohol users in Norway. All public and private residential institutions with an active contract with one of these regional health authorities were included in the surveys, with the exception of detoxification institutions.

The organization of the Norwegian healthcare system is divided into several levels, including the Regional Health Authorities, Health Enterprises, department, and section levels. The current measurements support adoption of patient-reported experiences in quality improvement work by health-care professionals, through quarterly reports at the lowest care level to all units with a sufficient number of responses. The lowest care level in this context refers to the section level. In any given period, there are approximately 110 active treatment centres participating in the surveys. Nationally, the number of discharges per year was approximately 15,000 in 2020 and 2021. The sections were in some instances institutions, which in other instances they were units organized within a hospital. Regional contact persons helped to compile the institution lists and establish contact persons at all care levels. Each participating unit had a project manager responsible for tasks that included disseminating information to the patients and employees, distributing login information to patients, and reporting back to the NIPH about the survey progress. Standardized guidelines for data collection were developed.

Continuous measurement results were obtained from adult inpatients (aged ≥ 16 years) who received interdisciplinary treatment for substance dependence. Project managers were instructed to include all patients as close as possible to discharge. To protect vulnerable patients, the institution's designated professional had the permission to exclude individual patients due to ethical considerations.

### Statistical analysis

In line with the international scientific literature, we analysed patient experience as a reflective construct [16, 20], and applied a combination of confirmatory factor analysis (CFA) and IRT to obtain information about the properties of the instrument. The previous psychometric testing of the PEQ-ITSD resulted in three empirically based scales with strong internal consistency, reliability, and validity, covering treatment and personnel, milieu, and outcome [13]. The PEQ-ITSD – CEM was developed and validated as part of a well-established national patient-experience program. The program's conceptual approach distinguishes between patient-reported experiences concerning non-clinical matters, patient-reported safety, and patient-reported outcomes, allowing for concurrent measurement of multiple components [21]. The conceptual approach draws inspiration from Donabedian's work [22] and establishes a connection between patient-reported experiences and the structures and processes of care, while patient satisfaction is considered an outcome. The three scales were interpreted within the context of the aforementioned terminology: the personnel and milieu scale are conceptually linked to patient-reported experiences, whereas the outcome scale is linked to patient-reported outcomes. Thus, the PEQ-ITSD – CEM has a clear and broad conceptual base, connecting evaluations from psychiatric patients to the tradition of patient-reported experiences [23], while simultaneously including an outcome scale that combines elements of the traditions of patient-satisfaction measurements and patient-reported outcomes [24–26].

Previous testing when validating the PIPEQ-CEM showed that comparing psychometric results from cross-sectional surveys and continuous measurements yielded similar results in both samples, despite differences in data collection approaches [15]. Since there were minor changes in the questionnaire, we did not anticipate substantial alterations in the factor structure. However, due to major changes in data collection from cross-sectional surveys and paper questionnaires to continuous electronic measurements, further validation of the instrument was necessitated to ensure that the data fit our hypothesized model which was based on the previous validation of the PEQ-ITSD. Therefore, the internal structure of the PEQ-ITSD – CEM was investigated using CFA. We aimed to determine if the data fit our hypothesized model which was based on the previous validation using an exploratory factor analysis (EFA) [13]. It was hypothesized that there was a second-order factor structure for the instrument, with experiences with treatment and personnel, milieu, and outcome as the lower-order factors, and inpatient experiences with treatment for substance dependence as the higher-order factors. We used diagonally weighted least squares (DWLS) to estimate the model parameters. Alternatives to the maximum likelihood should be used when continuous data do not conform to the normal distribution, or when some of the indicators are not interval-level (i.e. polytomous) [27]. When the ordered argument is used, the R package lavaan for CFA automatically switches to the robust variant and uses the DWLS to estimate the model parameters, but it then uses the full-weight matrix to compute robust standard errors, and a mean- and variance-adjusted test statistic. Observed variables were set to load onto a single latent variable with uncorrelated errors. Factor loading estimates between the first and second order variables were required to be > 0.35. The model fit was assessed using the root-mean-square error of approximation (RMSEA), goodness-of-fit index (GFI), comparative fit index (CFI), incremental fit index (IFI) and standardized root-mean-square residual (SRMR). A good fit was indicated by RMSEA ≤ 0.05, GFI ≥ 0.90, CFI ≥ 0.90, and SRMR < 0.08 [28]. IFI values are 0–1, with larger values indicating a better fit. A significant chi-square (χ^2^) statistic was expected since the sample size was > 250. Considering the sensitivity of the χ^2^ statistic to sample size, many other indices should be assessed to provide a more-holistic view of goodness of fit while accounting for sample size, model complexity and other considerations relevant to the respective study [27].

CFA were supplemented using IRT analyses since they provide a more-detailed description of the performance of each item, and useful during refinement to ensure that the best items were selected for the short-form PEQ-ITSD – CEM [17]. IRT analysis was applied to the items identified by the CFA to correspond to treatment and personnel, milieu, and outcome. The number of items made all three scales suitable for IRT analyses, which we applied to each scale separately. The instrument has polytomous response options, and the generalized partial credit model (GPCM) was used. Missing data were omitted from the analysis. The GPCM is one of the most flexible polytomous IRT models because it embodies fewer assumptions than other models such as partial credit and Bock’s nominal models or rating scale, and allows separate estimations of category-response and separate-discrimination parameters for each item [17]. The GPCM also has the advantage of allowing slope parameters to vary across items, and threshold parameters between response categories indicate the locations of the response options along the latent construct continuum. As a result of this flexibility, this model is more likely to fit data generated from patient-reported measures. Item performance was based on assessments of their discrimination (a) and location or difficulty (b). The null hypothesis for the S − χ^2^ item-fit statistic is that the item fits well, and a significant result indicates a poor fit [17]. However, these fit indices are sensitive to sample size, and even negligible differences can produce a result that indicate a poor fit in large samples. Because the sample of the present study was large, we chose to not report the S − χ^2^ statistic as a fitness indicator.

We aimed to ensure content coverage and selected the best-performing items from each of the scales. A shorter version of the PEQ-ITSD – CEM was developed by assessing the results of CFA and IRT analyses. The concordance between the long and short versions of the PEQ-ITSD – CEM instrument was assessed using intraclass correlation coefficients (ICCs). Statistical analyses were performed using SPSS software (version 28.0.1.0) and the R statistical software (version 4.0.2) with the lavaan, semPlot and mirt packages.

## Results

The data applied in the present study included responses from the first 3 years of continuous measurements made at residential institutions in Norway, starting in January 2020, and included 102 different sections and 2,850 patient responses. Respondent characteristics are listed in Table 1: 70.2% were male, 56.8% were 16–44 years old, the age at the onset of substance dependence was 28.5 years, 20.3% were married or living with a partner, 20.2% had received education to a university or college level, and 91.5% were born in Norway. The self-reported physical health was fair or poor in 31.8%; the corresponding proportion for self-reported mental health was 39.8%. The most frequently used substances prior to admission were alcohol (65.1%) and cocaine/amphetamines (34.9%). The stay duration for 49.6% of the participants was longer than 11 weeks, and 27.8% had three or more previous admissions. Compared to the distributions of gender, age, and length of stay reported in prior cross-sectional surveys from 2013, 2014, 2015, and 2017, as well as in activity data obtained from the Norwegian Patient Registry, the corresponding distributions in the current sample are similar. In 2021, the activity data from the Norwegian Patient Registry revealed that 66% of the patients were male, 52% were aged between 16 and 49, the most used substance prior to admission was alcohol, and the average length of stay was 43 days.

**Table 1:** Respondent characteristics (*n* = 2850)

	n	%
Sex		
Female	846	29.8
Male	1992	70.2
Age, years		
16–24	236	8.3
25–44	1380	48.5
45–66	1112	39.1
≥ 67	116	4.1
Age when substance dependence developed	2536	28.5 (mean)
Married or living with a partner		
Yes	574	20.3
No	2249	79.7
Highest level of education		
Primary school	838	29.6
Secondary school	1419	50.2
University or college	572	20.2
Country/region of birth		
Norway	2601	91.5
Nordic country other than Norway	59	2.1
Western Europe other than a Nordic country	20	0.7
Eastern Europe in EU	31	1.1
Eastern Europe not in the EU	14	0.5
Africa	40	1.4
Asia (including Turkey)	42	1.5
North America	15	0.5
South America or Central America	19	0.7
Oceania	1	0.0
Self-perceived physical health		
Excellent	172	6.1
Very good	621	21.9
Good	1142	40.3
Fair	638	22.5
Poor	264	9.3
Self-perceived mental health		
Excellent	138	4.9
Very good	542	19.1
Good	1029	36.3
Fair	777	27.4
Poor	352	12.4
Most frequently used drug/substance prior to this admission		
Alcohol	1854	65.1
Medication	758	27.5
Cannabis	884	31.0
Cocaine/amphetamine	996	34.9
Heroin/morphine	374	13.1
Other	272	9.5
Length of stay at this institution		
0–2 weeks	245	8.6
3–11 weeks	1186	41.7
3–6 months	853	30.0
7–12 months	387	13.6
More than 12 months	171	6.0
Previous admissions		
0	1026	36.1
1	637	22.4
2	387	13.6
3–5	494	17.4
> 5	295	10.4

Table 2 lists the mean values for the items of the three empirically based scales identified in the previous psychometric testing of the PEQ-ITSD covering treatment and personnel, milieu, and outcome. The mean score on the response scale from 1 to 5, where 5 was the best possible experience, was highest for item 25 (“Have you felt safe at the institution?”) (4.33), and lowest for item 19 (“Have you received help for physical health issues or illness”) (3.58).

**Table 2:** Item descriptives and mean scores of the PEQ-ITSD – CEM scales covering treatment and personnel, milieu, and outcome

		*n*	*Mean*^*a*^
	**Treatment and personnel**		
6	Have you had enough time for discussions and contact with the therapists/staff?	2835	4.07
7	Do you find that the therapists/staff have understood your situation?^b^	2835	4.19
8	Have you felt confident in the professional skills of the therapists/staff?	2832	4.21
9	Has one therapist/member of staff had primary responsibility for you?	2817	4.21
14	Has the information you have received about your treatment been satisfactory?	2799	3.95
15	Have you had influence on your treatment?	2810	3.95
16	Do you find that the treatment has been adapted to your needs?^b^	2825	3.91
19	Have you received help for physical health issues or illness?	2328	3.58
20	Have you received help for mental health issues?	2433	3.64
21	Have you had satisfactory access to a psychologist?	2535	3.63
22	Have you had satisfactory access to a medical doctor?	2751	3.65
31	Do you find that the therapists/staff have prepared you for the time after discharge?	2841	3.77
34	Do you find that the therapists/staff have helped you so that you can have a meaningful life after discharge?^b^	2650	3.86
33	Do you find that the therapists/staff have arranged continued treatment in the time after discharge?	2675	4.00
	**Milieu**		
4	Was the way you were welcomed to the institution satisfactory?	2844	4.28
25	Have you felt safe at the institution?^b^	2842	4.33
26	Has the institution arranged contact with other patients in a satisfactory manner?	2840	4.07
27	Has the range of activities available at the institution been satisfactory? ^b^	2836	3.60
29	Have the meals at the institution been satisfactory?	2839	4.05
30	Have you been satisfied with the level of privacy available?	2841	3.94
	**Outcome**		
13	Overall, to what extent have you benefitted from the treatment at the institution?^c^	2843	4.14
35	Overall, have the help and the treatment you have received at the institution been satisfactory?^b^	2835	4.13
36	Are the help and the treatment you are receiving at the institution helping you better understand your substance use issues?	2779	4.13
38	Are the help and the treatment you are receiving at the institution giving you confidence that life will be better after discharge?	2781	4.05
37	Are the help and the treatment you are receiving at the institution helping you better cope with your substance use issues? ^b^	2761	4.01

^a^Most items were scored on a 5-point response scale ranging from 1 (“not at all”) to 5 (“to a very large extent”)

^b^Items finally selected for the short form of the instrument

^c^Item scored on a 5-point response scale ranging from 1 (“no benefit”) to 5 (“very large benefit”)

**Fig. 1:**
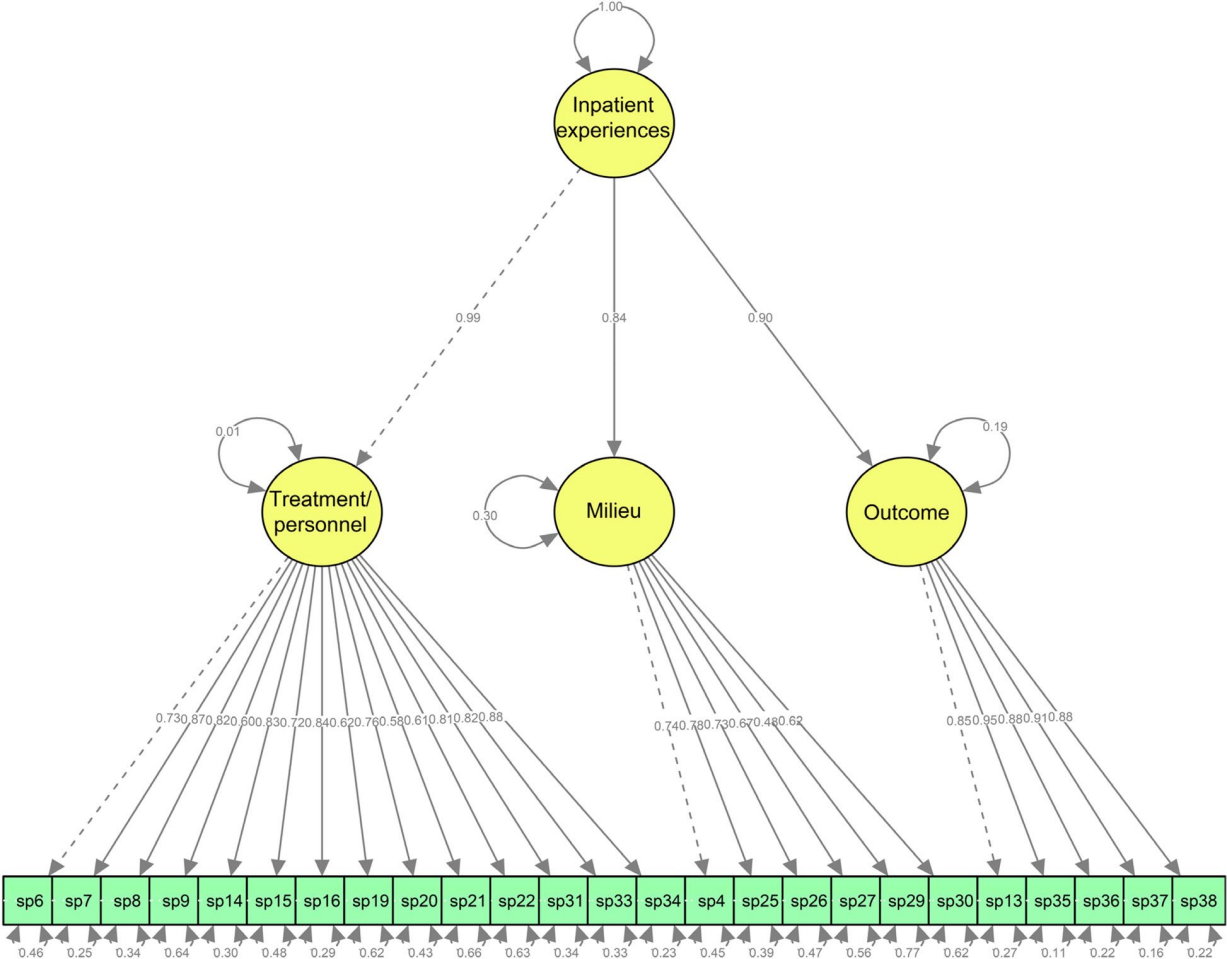
Confirmatory factor analysis model of the Patient Experiences Questionnaire for Interdisciplinary Treatment for Substance Dependence – Continuous Electronic Measurement (PEQ- ITSD – CEM)

The 3-factor solution from the previous EFA was tested using a CFA for the 25 items. The results are shown in Fig. 1, and indicate a reasonable model fit to the data (χ^2^ = 1934.53, *p* < 0.001, df = 272, RMSEA = 0.060, GFI = 0.99, CFI = 0.99, IFI = 0.99 and SRMR = 0.040). Inpatient experiences with interdisciplinary treatment for substance dependence were considered the second-order factor, and treatment and personnel, milieu, and outcome were considered the first-order factors. Experiences with treatment and personnel (γ = 0.99) and outcome (γ = 0.90) had the strongest relationships with the second-order factor, but experiences related to milieu (γ = 0.84) were also strongly related to the second-order factor.

Table 3 lists the results of the IRT analysis of the PEQ-ITSD – CEM and of the treatment and personnel, milieu, and outcome scales. None of the discrimination parameters exceeded 4.0. Item category thresholds varied across items, but were mostly concentrated below or around the average, indicating that the items were measured most accurately in the lower and middle sections of the scale.

**Table 3:** Parameter estimates from item response theory analysis according to the Patient Experiences Questionnaire for Interdisciplinary Treatment for Substance Dependence – Continuous Electronic Measurement (PEQ- ITSD – CEM)

		a	b1	b2	b3	b4
	**Treatment and personnel (*n* = 1790)**					
6	Have you had enough time for discussions and contact with the therapists/staff?	1.58	-2.58	-1.96	-0.99	0.39
7	Do you find that the therapists/staff have understood your situation?	2.51	-2.55	-1.84	-0.92	0.16
8	Have you felt confident in the professional skills of the therapists/staff?	2.00	-2.54	-2.10	-0.96	0.14
9	Has one therapist/member of staff had primary responsibility for you?	0.90	-2.12	-2.51	-1.52	-0.08
14	Has the information you have received about your treatment been satisfactory?	2.34	-2.22	-1.77	-0.75	0.68
15	Have you had influence on your treatment?	1.41	-2.28	-2.31	-0.78	0.58
16	Do you find that the treatment has been adapted to your needs?	2.30	-2.20	-1.76	-0.56	0.57
19	Have you received help for physical health issues or illness?	0.84	-2.05	-1.74	-0.17	0.88
20	Have you received help for mental health issues?	1.34	-2.05	-1.44	-0.27	0.75
21	Have you had satisfactory access to a psychologist?	0.64	-1.80	-1.31	-0.73	0.56
22	Have you had satisfactory access to a medical doctor?	0.79	-3.16	-1.25	-0.41	0.96
31	Do you find that the therapists/staff have prepared you for the time after discharge?	1.90	-1.95	-1.51	-0.49	0.79
33	Do you find that the therapists/staff have arranged continued treatment in the time after discharge?	1.79	-1.85	-1.66	-0.68	0.34
34	Do you find that the therapists/staff have helped you so that you can have a meaningful life after discharge?	2.37	-1.93	-1.42	-0.52	0.56
	**Value ranges**	0.64 to 2.51	–1.80 to –3.16	–1.25 to –2.51	–0.17 to –1.52	0.08 to 0.96
	**Milieu (*n* = 2809)**					
4	Was the way you were welcomed to the institution satisfactory?	1.23	-2.57	-2.34	-1.85	0.11
25	Have you felt safe at the institution?	1.94	-3.01	-2.12	-1.63	0.06
26	Has the institution arranged contact with other patients in a satisfactory manner?	1.79	-2.38	-2.06	-1.07	0.46
27	Has the range of activities available at the institution been satisfactory?	0.92	-2.68	-1.59	-0.37	1.17
29	Have the meals at the institution been satisfactory?	0.61	-2.93	-2.56	-1.52	0.13
30	Have you been satisfied with the level of privacy available?	1.00	-2.11	-2.00	-1.11	0.60
	**Value ranges**	0.61 to 1.94	–2.11 to –3.01	–1.59 to –2.56	–0.37 to –1.85	0.06 to 1.17
	**Outcome (*n* = 2686)**					
13	Overall, to what extent have you benefitted from the treatment at the institution?^†^	2.03	-2.88	-2.16	-1.06	0.24
35	Overall, have the help and the treatment you have received at the institution been satisfactory?	2.49	-2.80	-2.08	-1.11	0.38
36	Are the help and the treatment you are receiving at the institution helping you better understand your substance use issues?	2.67	-2.27	-1.85	-0.85	0.13
37	Are the help and the treatment you are receiving at the institution helping you better cope with your substance use issues?	4.46	-2.36	-1.73	-0.67	0.39
38	Are the help and the treatment you are receiving at the institution giving you confidence that life will be better after discharge?	3.17	-2.39	-1.89	-0.75	0.34
	**Value ranges**	2.03 to 4.46	–2.27 to –2.88	–1.73 to –2.16	–0.67 to –1.11	0.13 to 0.39

a: discrimination; b1–b4: thresholds

**Fig. 2:**
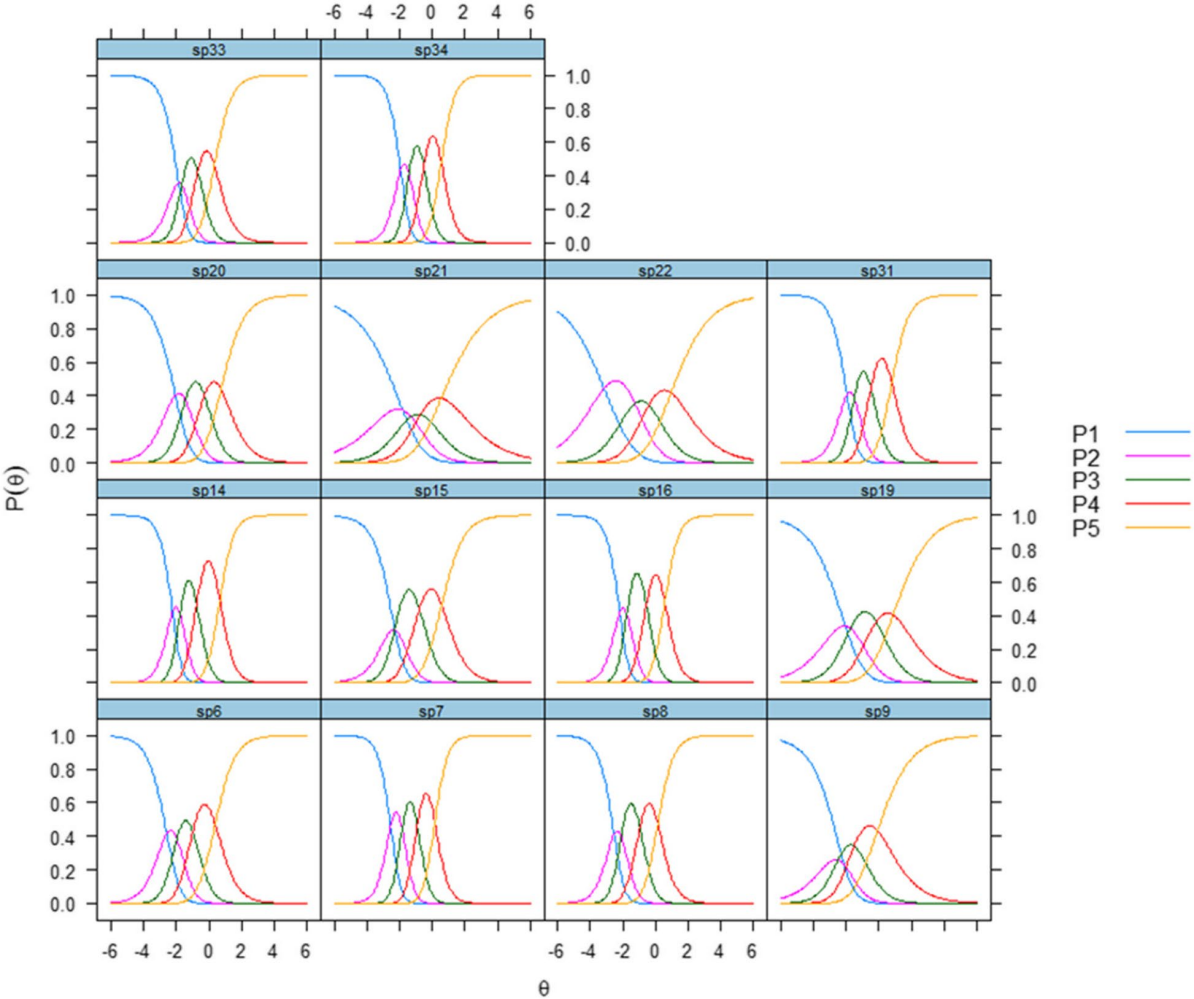
Categorical response curves of the Patient Experiences Questionnaire for Interdisciplinary Treatment for Substance Dependence – Continuous Electronic Measurement (PEQ- ITSD – CEM), Treatment and personnel P1-P5 denote the response scale: P1 = "not at all," P2 = "to a small extent," P3 = "to some extent," P4 = "to a large extent," P5 = "to a very large extent."

Item results for the treatment and personnel scale indicated that discrimination parameters varied from 0.64 for item 21 (“Have you had satisfactory access to a psychologist?”) to 2.51 for item 7 (“Do you find that the therapists/staff have understood your situation?”) (Table 3). The first, second, third and fourth item thresholds varied from –1.80 to –3.16, from –1.25 to –2.51, from –1.17 to 1.52 and from 0.08 to 0.96, respectively. The categorical response curves (CRCs) in Fig. 2 visualize the discrimination and category thresholds of the items, and further illustrates that the second response category had questionable value for several of the items, particularly for items 9, 15 and 33.

**Fig. 3:**
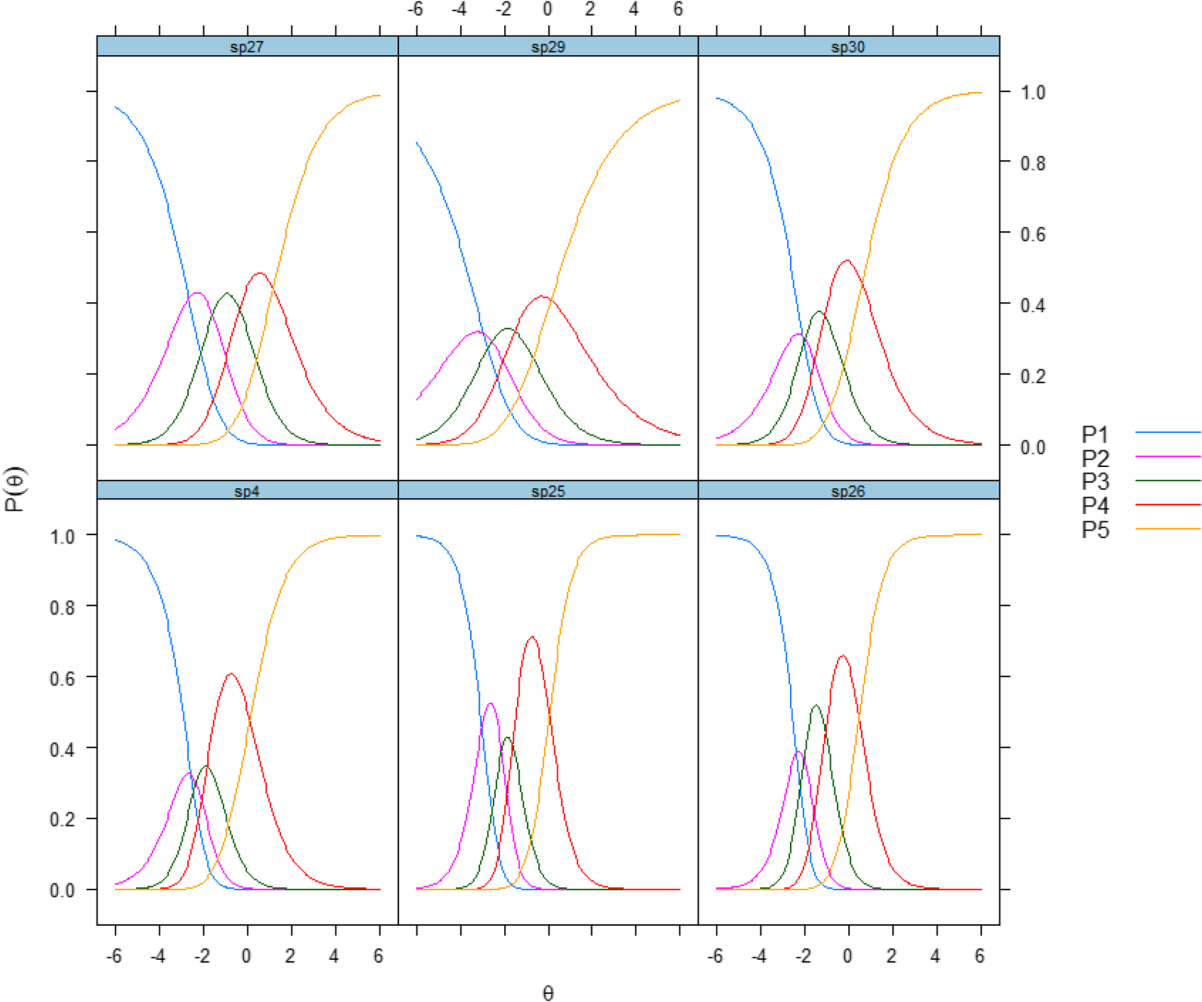
Categorical response curves of the Patient Experiences Questionnaire for Interdisciplinary Treatment for Substance Dependence – Continuous Electronic Measurement (PEQ- ITSD – CEM), Milieu P1-P5 denote the response scale: P1 = "not at all," P2 = "to a small extent," P3 = "to some extent," P4 = "to a large extent," P5 = "to a very large extent."0

Items 25 (“Have you felt safe at the institution?”) and 26 (“Has the institution arranged contact with other patients in a satisfactory manner?”) on the milieu scale were those with the best discriminative abilities (Table 3). The first, second, third, and fourth item thresholds varied from –2.11 to –3.01, from –1.59 to –2.56, from –0.37 to –1.85 and from 0.06 to 1.17, respectively. The CRCs in Fig. 3 illustrate that the second response category had questionable value for item 30.

**Fig. 4:**
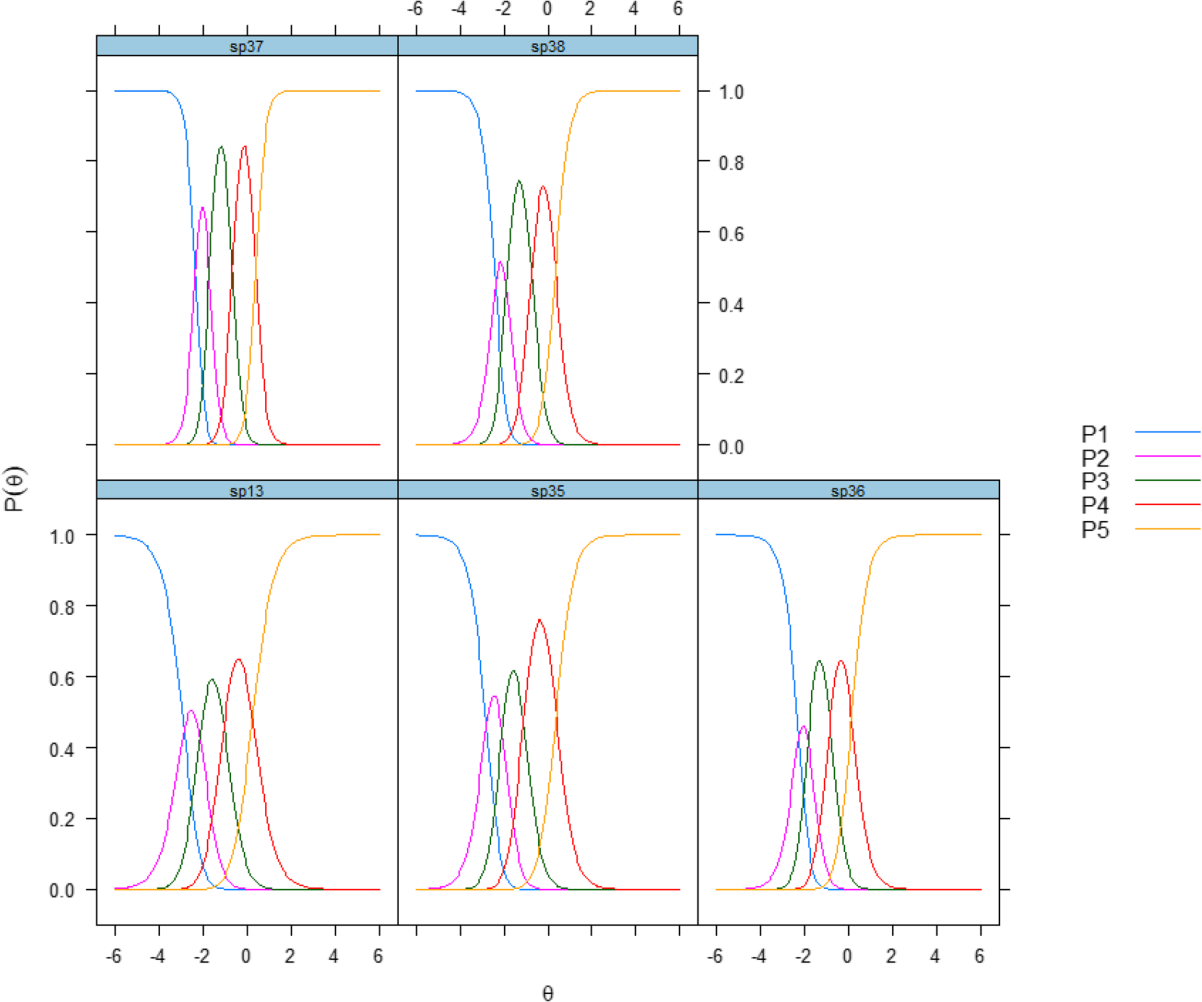
Categorical response curves of the Patient Experiences Questionnaire for Interdisciplinary Treatment for Substance Dependence – Continuous Electronic Measurement (PEQ- ITSD – CEM), Outcome P1-P5 denote the response scale: P1 = "not at all," P2 = "to a small extent," P3 = "to some extent," P4 = "to a large extent," P5 = "to a very large extent."

The outcome items with the highest discrimination level were items 37 (4.46; “Are the help and the treatment you are receiving at the institution helping you better cope with your substance use issues?”) and item 38 (3.17; “Are the help and the treatment you are receiving at the institution giving you confidence that life will be better after discharge?”). The first, second, third and fourth item thresholds varied from –2.27 to –2.88, from –1.73 to –2.16, from –0.67 to –1.11 and from 0.13 to –0.39, respectively. Figure 4 shows the discrimination and category thresholds for items in the scale.

A secondary objective was to select items that ensured an even distribution across various locations to develop a short version. We selected seven items after assessing factor loadings and item performance, and aimed to ensure coverage of the three scales.

Regarding the treatment and personnel scale, the items with the strongest connections to the latent factor in the CFA were item 34 (“Do you find that the therapists/staff have helped you so that you can have a meaningful life after discharge?”), item 7 and item 16 (“Do you find that the treatment has been adapted to your needs?”) (Fig. 1). IRT indicated that items 7 and 34 had the best discriminative abilities (Table 3). Those with the weakest connections to the latent factor in the CFA were items 21 and 9 (“Has one therapist/member of staff had primary responsibility for you?”) (Fig. 1). We selected items 34, 7 and 16 for the short form of the instrument, which was also aided through examination of the information function curves shown in Fig. 2. The CRCs indicated that the response categories of these items covered a wide range of theta (Fig. 2).

For the milieu scale, the results from the CFA showed that the items with the strongest connections to the latent factor were items 25 and 4 (“Was the way you were welcomed to the institution satisfactory?”) (Fig. 1). The items with the best discriminative abilities were 25 and 26, with slope estimates of 1.94 and 1.79, respectively (Table 3). Item 27 (“Has the range of activities available at the institution been satisfactory?”) measured at a higher location to the latent construct than the other items. The CRCs for the items are shown in Fig. 3. We selected items 25 and 27 for the short form of the instrument to ensure content coverage and relevant topics.

Regarding outcome, the items with best discriminative abilities in the IRT analysis (Table 3) were items 37 and 38. Figure 1 shows that items 35 (“Overall, have the help and the treatment you have received at the institution been satisfactory?”) and 37 had strongest connections to the latent construct. The CRCs in Fig. 4 indicated that the response categories of all items of the outcome scale covered a wide range of theta (Fig. 1), but we selected items 37 and 35 for the short form of the instrument. Item 35 was selected over item 38 in order to maximize information with minimal content overlap.

The ICC between the 25-item PEQ-ITSD – CEM instrument and its 7-item short version was 0.97 (*p* < 0.001). The ICCs between the full treatment and personnel, milieu, and outcome scales and the selected items were 0.95 (*p* < 0.001), 0.91 (*p* < 0.001) and 0.98 (*p* < 0.001), respectively.

## Discussion

The main aim of this study was to determine the psychometric properties of the PEQ-ITSD – CEM, which is used in currently used for large-scale measurements in Norway. The secondary objectives were to provide more information on the performance of its items, and to develop a short version of the instrument.

The psychometric testing of the instrument provided good evidence for data quality and internal consistency. The PEQ-ITSD – CEM is multidimensional and comprises three scales based on both empirical and theoretical assumptions, and covers the evaluations of treatment and personnel, milieu, and outcome. The domains in the present study corresponded to those identified in reviews of PREMs in mental health care that highlight the importance of interpersonal relationships, respect and dignity, access and care coordination, information, psychological care, care environment and outcomes [16, 29–31].

The psychometric analyses results revealed a stable and interpretable scale structure that supported the previous validation of the instrument [13]. Minor changes in item composition were found, partly due to the change in data collection from cross-sectional surveys to continuous measurements. The low proportion of omitted answers suggested good acceptability, and the few responses in the “not applicable” option indicated that the questions were relevant to most of the patients. Patients were involved in the development of the PEQ-ITSD – CEM, including identifying critical aspects of care, and the instrument comprises factual questions that directly address a broad range of specific domains.

Furthermore, our study found that the PEQ-ITSD – CEM could be reduced from 25 items to a 7-item short version that consists of items from the three scales. The short version provides an efficient approach for brief yet comprehensive measurements, allowing participants to answer fewer questions without reducing the measurement precision. This study applied both CTT and IRT methods to evaluate survey items, and the results provided useful and different insights into the performance of the PEQ-ITSD – CEM instrument. However, data collection strategies that focus solely on reducing burden may result in the loss of important information and reduced representativeness of patient survey responses [32]. Selecting items entirely based on statistics can identify those that can be improved on in theory, but this approach may have little relevance to clinical practice (both for patients and health-care providers) [5]. Previous national surveys involving both quantitative and qualitative data support the selection of items for the short-form PEQ-ITSD – CEM. However, future research should include additional inputs from health-care professionals and patients.

Providing continuous web-based surveys avoids a time gap between the collection and reporting of data, making the results suitable for monitoring performance in real time and helping the residential institutions to identify areas where quality improvements are required [33]. Better experiences are associated with higher adherence levels to recommended prevention and treatment processes, better clinical outcomes, better patient safety and less health-care utilization [3]. Future research should explore the use of these data by health-care professionals in implementing improvements, followed by subsequent patient-experience surveys to monitor changes over time.

We were aware of few national continuous measurements of the experiences of patients in substance-dependence treatment. The NIPH has established contact at all levels, and all institutions have been instructed to establish their own data collection routines. The aim is to include all patients who meet the eligibility criteria to obtain representative data, but many are still not invited to participate in the survey. In future surveys we will combine the existing on-site approach with a post-discharge one starting with a pilot in November 2023. In the latter, we will obtain background data from the Norwegian Patient Registry, which will enable the enrolment of all patients discharged from residential institutions, and the use of non-response analysis and case-mix adjustments. We will also explore the potential for constructing and reporting quality indicators based on the PEQ-ITSD – CEM in Norway.

### Strengths and limitations

Web-based surveys are a feasible and time-effective approach to collecting survey data, and address the pressure to reduce the costs of survey administration. Another strength of this study was that it was performed by a third party (the NIPH) that does not provide health care.

Limitations of this study need to be acknowledged. First, employees at psychiatric institutions have raised concerns regarding the cognitive abilities and digital literacy of patients in mental health care, and emphasized the need for a shorter questionnaire, or even the use of paper questionnaires. These sorts of problems have not been reported among employees at residential institutions, but it is possible that the web-based surveys excluded those with poor digital literacy.

Second, selection bias may have occurred due to the providers of the services being responsible for inviting the participants. Employees might have influenced patient responses despite being instructed not to influence them in any way.

Third, no background data on the patients were collected from patient registries in the current measurements. Consequently, there was no way to ascertain whether a patient had been included multiple times. Without measures to control for more than one response for each individual we cannot conclude that all observations are independent. In practice, we believe that the total number of persons with more than one response is small. Many patients will not have the opportunity to answer the questionnaire more than one time because they are not readmitted. In addition, readmitted patients with a prior response will struggle even more to engage and be motivated to answer the same questionnaire again. However, even with a small number it means that results for providers must be interpreted as average patient experience scores with potential multiple answers for some patients because of readmissions. It also means that results at more aggregate levels will include potentially multiple answers for some individuals both for readmissions to the same units and for admissions/readmissions to other units. Since each inpatient stay might vary in terms of structure (unit/provider factors), process and outcome we believe multiple responses from individuals are the preferred measurement model, so the challenge in our context is the fact that we cannot quantify and adjust for the extent of multiple responses. From a statistical point, it means that analysis requiring independent observations must be conducted with caution and with appropriate discussion of statistical uncertainty. We believe the magnitude and thus the practical and statistical effects are small, but to reduce this uncertainty and improve data quality the continuous, digital measurements will be non-anonymous from the pilot in November 2023. This means that we will be able to both quantify the extent of multiple responses from individuals over time and adjust for it in sampling and in statistical analysis. The inclusion of the same individuals over time will be rule-based, e.g., only include individuals with readmissions 1–2 months after being enrolled the first time. An important consideration for deciding the proper time interval will be respondent burden, to avoid unnecessary burden on top of the symptom burden for these patients.

Fourth, the validation process built upon previous psychometric testing of the PEQ-ITSD, where items with missing data or "not applicable" responses exceeding > 20% were excluded. Excluding certain items from the analysis can inadvertently bias results by focusing on easily observed or reported variables, potentially favouring a specific subgroup. This can reduce the findings' generalizability and affect the validity of measures. However, in healthcare quality monitoring it is crucial for external indicators to reflect the majority of patients' experiences. If many respondents find certain questions irrelevant, those questions may not be suitable as generic external quality indicators, even though they remain valuable for specific purposes. Fortunately, issues related to item missing were not a major problem in the current study [13].

Fifth, depending on the specific context and purpose for which the short form is to be used, researchers and practitioners may need to consider employing the full instrument or selectively including items deemed particularly crucial [19], e.g., including more or all outcome items if outcome is considered paramount. The abbreviated version of the questionnaire will not be sufficiently comprehensive in many applications, which means that it primarily is meant for applications where respondent burden is deemed especially important.

## Conclusions

The PEQ-ITSD – CEM comprises three scales with satisfactory internal consistency, reliability and validity. The instrument can be used to assess the experiences of patients in interdisciplinary treatment for substance dependence in Norway, as well as in other countries with similar health-service organizations. The seven-item short version can be applied where respondent burden is a major concern. Future research should include the priorities of health-care professionals and patients. When conducting continuous measurements, the validation process should also be interpreted as continuous rather than a single procedure.

### Supplementary Information

**Additional file 1.**

## Data Availability

The data sets generated and/or analysed during the current study are not publicly available due to the need to protect personal data but are available from the corresponding author on reasonable request.

## Abbreviations

PREM: Patient-reported experience measure
NIPH: Norwegian Institute of Public Health
PEQ-ITSD: Patient Experiences Questionnaire for Interdisciplinary Treatment for Substance Dependence
SUD: Substance Use Disorder
PIPEQ – CEM: Psychiatric Inpatient Patient Experience Questionnaire – Continuous Electronic Measurement
CTT: Classical test theory
IRT: Item response theory
PEQ-ITSD – CEM: Patient Experiences Questionnaire for Interdisciplinary Treatment for Substance Dependence – Continuous Electronic Measurement
CFA: Confirmatory factor analysis
EFA: Exploratory factor analysis
DWLS: Diagonally weighted least squares
RMSEA: Root-mean-square error of approximation
GFI: Goodness-of-fit index
CFI: Comparative fit index
IFI: Incremental fit index
SRMR: Standardized root-mean-square residual
χ^2^: Chi-square
GPCM: Generalized partial credit model
ICC: Intraclass correlation coefficients
CRC: Categorical response curves
