# Supplementary material for: Reliability and validity of the Patient Experiences Questionnaire for Interdisciplinary Treatment for Substance Dependence – Continuous Electronic Measurement (PEQ-ITSD – CEM)
Source: BMC Health Serv Res. 2024 Jan 4;24:26. doi: 10.1186/s12913-023-10506-7 (PMC10768463; doi:10.1186/s12913-023-10506-7)
Supplement: Supplementary file 1 — Additional file 1. [file 12913_2023_10506_MOESM1_ESM.pdf]

NIPH
Norwegian Institute of Public Health

What are your experiences with your inpatient stay in an institution for substance abuse treatment?

The purpose of this survey is to improve the services for patients within substance abuse treatment. We are interested in your experiences with the institution where you are now a patient.

How to fill in the form: Enter an X in the centre of the appropriate box.
Like this: ☒ Not like this: ☐

A little about your background

1. Which substance did you use most before your current admission? *You can choose several options.*

Alcohol	Medication	Cannabis/ hash	Cocaine/ amphetamines	Heroin/ morphine substances	Other	Not applicable
☐	☐	☐	☐	☐	☐	☐

2. How long have you been an inpatient at this institution?

0-2 weeks	3-11 weeks	3-6 months	7-12 months	More than 12 months
☐	☐	☐	☐	☐

Reception and waiting time

3. Did you receive information about the institution's rules and routines when you arrived?

Not at all	To a small extent	To some extent	To a large extent	To a very large extent
☐	☐	☐	☐	☐

4. Was the way you were welcomed to the institution satisfactory?

Not at all	To a small extent	To some extent	To a large extent	To a very large extent
☐	☐	☐	☐	☐

5. Did you have to wait to be admitted to the institution?

No	Yes, but not long	Yes, quite long	Yes, far too long
☐	☐	☐	☐

Therapists/staff

Keep the therapists and staff at the institution in mind when you answer the following questions.

	Not at all	To a small extent	To some extent	To a large extent	To a very large extent	Not applicable
6. Have you had enough time for discussions and contact with the therapists/staff?	☐	☐	☐	☐	☐	☐
7. Do you find that the therapists/staff have understood your situation?	☐	☐	☐	☐	☐	☐
8. Have you felt confident in the professional skills of the therapists/staff?	☐	☐	☐	☐	☐	☐
9. Has one therapist/member of staff had primary responsibility for you?	☐	☐	☐	☐	☐	☐
10. To what extent have you been met with courtesy and respect?	☐	☐	☐	☐	☐	☐

	No, never	Yes, once	Yes, a few times	Yes, many times
11. Have you been patronised or insulted by the therapists/staff?	☐	☐	☐	☐

Treatment

12. To what extent have you benefitted from the following types of treatment at the institution: Choose "*Not applicable*" for treatments you do not have experience with.

	No benefit	Small benefit	Some benefit	Large benefit	Very large benefit	Not applicable
Treatment in groups?	☐	☐	☐	☐	☐	☐
Conversations with one therapist?	☐	☐	☐	☐	☐	☐
Treatment with medication?	☐	☐	☐	☐	☐	☐

	No benefit	Small benefit	Some benefit	Large benefit	Very large benefit
13. Overall, to what extent have you benefitted from the treatment at the institution?	☐	☐	☐	☐	☐

		Not at all	To a small extent	To some extent	To a large extent	To a very large extent	Not applicable
14.	Has the information you have received about your treatment been satisfactory?	☐	☐	☐	☐	☐	☐
15.	Have you had influence on your treatment?	☐	☐	☐	☐	☐	☐
16.	Do you find that the treatment has been adapted to your needs?	☐	☐	☐	☐	☐	☐
17.	Have you had the opportunity to give feedback on your treatment after talking to a therapist?	☐	☐	☐	☐	☐	☐
18.	Have you been involved in the choice of treatment you are currently receiving?	☐	☐	☐	☐	☐	☐

		Not at all	To a small extent	To some extent	To a large extent	To a very large extent	Not applicable
19.	Have you received help for physical health issues or illness?	☐	☐	☐	☐	☐	☐
20.	Have you received help for mental health issues?	☐	☐	☐	☐	☐	☐

		Not at all	To a small extent	To some extent	To a large extent	To a very large extent	Not applicable
21.	Have you had satisfactory access to a psychologist?	☐	☐	☐	☐	☐	☐
22.	Have you had satisfactory access to a medical doctor?	☐	☐	☐	☐	☐	☐

		Yes	No	Not applicable
23.	Has your physical health been examined by a medical doctor during this stay (e.g. blood tests, listening to heart, heart rate and weight)?	☐	☐	☐
24.	Have your teeth been assessed by a medical doctor during this stay?	☐	☐	☐

Environment and activities

	Not at all	To a small extent	To some extent	To a large extent	To a very large extent
25. Have you felt safe at the institution?	☐	☐	☐	☐	☐
26. Has the institution arranged contact with other patients in a satisfactory manner?	☐	☐	☐	☐	☐
27. Has the range of activities available at the institution been satisfactory?	☐	☐	☐	☐	☐
28. Has the institution given you the opportunity to be physically active (e.g. walking, jogging, exercising)?	☐	☐	☐	☐	☐
29. Have the meals at the institution been satisfactory?	☐	☐	☐	☐	☐
30. Have you been satisfied with the level of privacy available?	☐	☐	☐	☐	☐

Preparing for the time after discharge

The following questions are about preparations for the time after discharge.

	Not at all	To a small extent	To some extent	To a large extent	To a very large extent	Not applicable
31. Do you find that the therapists/staff have prepared you for the time after discharge?	☐	☐	☐	☐	☐	
32. Do you find that the therapists/staff have helped you with *practical issues* for the time after discharge (e.g. housing, finances, work/school)?	☐	☐	☐	☐	☐	☐
33. Do you find that the therapists/staff have arranged continued *treatment* in the time after discharge?	☐	☐	☐	☐	☐	☐
34. Do you find that the therapists/staff have helped you so that you can have a meaningful life after discharge?	☐	☐	☐	☐	☐	☐

35. Overall, have the help and the treatment you have received at the institution been satisfactory?

Not at all	To a small extent	To some extent	To a large extent	To a very large extent
☐	☐	☐	☐	☐

36. Are the help and the treatment you are receiving at the institution helping you better *understand* your substance abuse issues?

Not at all	To a small extent	To some extent	To a large extent	To a very large extent	Don't know
☐	☐	☐	☐	☐	☐

37. Are the help and the treatment you are receiving at the institution helping you better *cope with* your substance abuse?

Not at all	To a small extent	To some extent	To a large extent	To a very large extent	Don't know
☐	☐	☐	☐	☐	☐

38. Are the help and the treatment you are receiving at the institution giving you confidence that life will be better after discharge?

Not at all	To a small extent	To some extent	To a large extent	To a very large extent	Don't know
☐	☐	☐	☐	☐	☐

39. Do you find that the therapists/staff have cooperated well with your relatives?

Not at all	To a small extent	To some extent	To a large extent	To a very large extent	Not applicable
☐	☐	☐	☐	☐	☐

40. To what extent did you feel pressured/ forced by others to be admitted?

Not at all	To a small extent	To some extent	To a large extent	To a very large extent	Not applicable
☐	☐	☐	☐	☐	☐

41. Do you believe that you have been incorrectly treated in any way (according to your own judgement)?

Not at all	To a small extent	To some extent	To a large extent	To a very large extent	Not applicable
☐	☐	☐	☐	☐	☐

Previous admissions to substance abuse treatment institutions

The following questions relate to previous admissions to substance abuse treatment institutions, with the exception of units only for detox.

42. Have you been admitted to a substance abuse treatment institution prior to this admission?

No	Yes, once	Yes, 2 times	Yes, 3-5 times	Yes, more than 5 times
☐	☐	☐	☐	☐

43. If you have previously been admitted, did you find the follow-up/aftercare after discharge satisfactory? (Think about the most recent admission if you have been admitted several times.)

Not at all	To a small extent	To some extent	To a large extent	To a very large extent	Not applicable
☐	☐	☐	☐	☐	☐

44. If you have previously been admitted, was the last admission at this institution?

Yes	No	Not applicable
☐	☐	☐

Help from the municipality where you live

45. Do you find that the municipality where you live provides follow-up during your admission (for example, a substance abuse consultant, GP/family doctor or the team responsible for your care)?

Not at all	To a small extent	To some extent	To a large extent	To a very large extent	Not applicable
☐	☐	☐	☐	☐	☐

46. If you have previously received help from the municipality where you live, has the help been satisfactory overall?

Not at all	To a small extent	To some extent	To a large extent	To a very large extent	Not applicable/ have not received help
☐	☐	☐	☐	☐	☐

47. If you have previously received help from the municipality where you live, who or which unit has been most important for you?
You can choose several options.

Substance abuse consultant	Team responsible for your care	Follow-up services at home	24-hour service	GP/family doctor	Other	Not applicable/ have not received help
☐	☐	☐	☐	☐	☐	☐

48. If you reply “Other” to question 47, please specify the unit. ______________________

Background information

49. Are you female or male?

Female	Male
☐	☐

50. How old are you?

16-24	25-44	45-66	67 or older
☐	☐	☐	☐

51. How old were you when you first had substance abuse problems (enter age)?

☐☐

52. Are you married/living with a partner?

Yes	No
☐	☐

53. What is your highest level of education?

Compulsory primary school (grades 1-10)	Upper secondary school	College/ university
☐	☐	☐

54. Where were you born?

Norway	In a Nordic country (other than Norway)	Western Europe (other than a Nordic country)	EU country in Eastern Europe	Eastern Europe (not a country in the EU)	Africa	Asia (including Turkey)	North America	South America or Central America	Oceania
☐	☐	☐	☐	☐	☐	☐	☐	☐	☐

55. Do you have an individual plan for treatment/care? (Anyone in need of long-term and coordinated health and social services is entitled to an individual plan.)

Yes	No	Don't know
☐	☐	☐

56. If you have an individual plan, are you satisfied with this plan?

Not at all	To a small extent	To some extent	To a large extent	To a very large extent	Not applicable
☐	☐	☐	☐	☐	☐

57. How would you describe your *physical* health?

Excellent	Very good	Good	Fair	Poor
☐	☐	☐	☐	☐

58. How would you describe your *mental* health?

Excellent	Very good	Good	Fair	Poor
☐	☐	☐	☐	☐

Feel free to write more about your experiences with your stay at the substance abuse treatment institution:

Feel free to write more about your past experiences with help from the municipality where you live (e.g. in relation to housing, finances, work/school and health and care services):

Thank you for taking the time to complete the survey!
